# Single-cell RNA sequencing reveals B cell–related molecular biomarkers for Alzheimer’s disease

**DOI:** 10.1038/s12276-021-00714-8

**Published:** 2021-12-08

**Authors:** Liu-Lin Xiong, Lu-Lu Xue, Ruo-Lan Du, Rui-Ze Niu, Li Chen, Jie Chen, Qiao Hu, Ya-Xin Tan, Hui-Fang Shang, Jia Liu, Chang-Yin Yu, Ting-Hua Wang

**Affiliations:** 1 Department of Anesthesiology, The Affiliated Hospital of Zunyi Medical University, Zunyi, 563000 Guizhou China; 2 Yunnan Key Laboratory of Primate Biomedical Research, Institute of Primate Translational Medicine, Kunming University of Science and Technology, Kunming, 650504 Yunnan China; 3 State Key Laboratory of Biotherapy, West China Hospital, Sichuan University, Chengdu, 610041 Sichuan China; 4 Laboratory Animal Department, Kunming Medical University, Kunming, 650031 Yunnan China; 5 Institute of Neurological Disease, Translational Neuroscience Center, West China Hospital, Sichuan University, Chengdu, 610041 Sichuan China; 6 School of Anesthesiology , Southwest Medical University, Luzhou, 646000 Sichuan China; 7 Department of Neurology, West China Hospital, Sichuan University, Chengdu, 610041 Sichuan China; 8 Department of Neurology, Affiliated Hospital of Zunyi Medical University, Zunyi, 563000 Guizhou China

**Keywords:** Cell death in the nervous system, Neurotrophic factors

## Abstract

In recent years, biomarkers have been integrated into the diagnostic process and have become increasingly indispensable for obtaining knowledge of the neurodegenerative processes in Alzheimer’s disease (AD). Peripheral blood mononuclear cells (PBMCs) in human blood have been reported to participate in a variety of neurodegenerative activities. Here, a single-cell RNA sequencing analysis of PBMCs from 4 AD patients (2 in the early stage, 2 in the late stage) and 2 normal controls was performed to explore the differential cell subpopulations in PBMCs of AD patients. A significant decrease in B cells was detected in the blood of AD patients. Furthermore, we further examined PBMCs from 43 AD patients and 41 normal subjects by fluorescence activated cell sorting (FACS), and combined with correlation analysis, we found that the reduction in B cells was closely correlated with the patients’ Clinical Dementia Rating (CDR) scores. To confirm the role of B cells in AD progression, functional experiments were performed in early-stage AD mice in which fibrous plaques were beginning to appear; the results demonstrated that B cell depletion in the early stage of AD markedly accelerated and aggravated cognitive dysfunction and augmented the Aβ burden in AD mice. Importantly, the experiments revealed 18 genes that were specifically upregulated and 7 genes that were specifically downregulated in B cells as the disease progressed, and several of these genes exhibited close correlation with AD. These findings identified possible B cell-based AD severity, which are anticipated to be conducive to the clinical identification of AD progression.

## Alzheimer’s disease: A new biomarker for disease progression?

Molecular tests built around analyzing B cells, a specialized type of immune cell, could aid in the diagnosis of Alzheimer’s disease. Liu-Lin Xiong from the Affiliated Hospital of Zunyi Medical University, China, and coworkers used single-cell RNA sequencing to profile gene activity in individual peripheral blood mononuclear cells from people with and without Alzheimer’s disease. They discovered that people with Alzheimer’s, especially those with more advanced disease, had lower levels of circulating B cells than healthy subjects. Twenty-five specific genes in the B cells were expressed at significantly higher or lower levels as the disease progressed. The researchers found similar results regarding B cells and Alzheimer’s progression in mouse models, and showed that massive depletion of B cells in the early onset was associated with accelerated cognitive decline and increased accumulation of sticky brain plaques.

## Introduction

Alzheimer’s disease (AD) has long been one of the great challenges in medicine and imposes a constant burden on our aging population. Recent statistics show that approximately 50 million people worldwide suffer from AD or some other form of dementia^1^. The World Health Organization has estimated that the total number of people with dementia worldwide will reach 82 million by 2030 and 152 million by 2050^2^. Of the top 10 leading causes of death based on United States cancer statistics, cardiovascular disease ranks first, tumors rank second and AD ranks sixth^3^. AD, an insidious progressive neurodegenerative disease, is clinically characterized by cognitive dysfunction, psychiatric symptoms, behavioral disorders, and even a gradual decline in the ability to carry out activities of daily living^4,5^. Considered a heterogeneous disease, the disease may be attributed to family history, head trauma, low educational level, thyroid disease, too high or too low maternal reproductive age, and viral infection^6^. At present, AD can be diagnosed by neuropsychological tests, hematological examinations, neuroimaging examinations, electroencephalograms, cerebrospinal fluid tests, genetic tests and so on^7,8^. In the past few years, the International Working Group (IWG) has integrated biomarkers into the diagnostic process, and in 2014, the IWG began to divide AD biomarkers into diagnostic markers and progression markers to cover all stages of the disease (from asymptomatic status to the most severe stage of dementia)^9,10^.

Human blood, which can contain crucial biomarkers, has been used to reflect the physiological and pathological condition of patients. The key role of peripheral blood in the diagnosis of AD has been identified in previous studies^11,12^, and peripheral blood mononuclear cells (PBMCs) have been reported to play an active role in a variety of neurodegenerative events^13^. PBMCs consist of multiple cell subsets, which are commonly divided into myeloid and lymphoid cells; myeloid cells comprise monocytes and their descendants, along with granulocytes such as neutrophils and basophils, while lymphoid cells primarily include T cells, B cells and NK cells^14^. Total PBMC-based genome-wide expression measurements can detect the proportions of various cell subpopulations in a given sample^13,15^, whereas the lesser transcriptional alterations in some subsets cannot be determined by single-cell RNA sequencing (RNA-seq) of bulk PBMCs^16^. Thankfully, the reliable measurement of transcriptional heterogeneity has been made possible by microfluidic control of cell capture and preparation of RNA-seq libraries from single individual cells, which has been successfully applied to identify cell subsets and critical biomarkers in multiple distinct biological settings, such as neurogenesis, tumorigenesis and embryonic development^17–20^. Single-cell RNA-seq possesses the capacity to identify specific biomarkers so that multiple candidate biomarkers can be selected for diagnostic and prognostic use.

In the current study, differentially abundant cell subpopulations among PBMCs and differentially expressed genes in B cells were identified in 4 AD patients and 2 controls by single-cell sequencing analysis. Moreover, we collected PBMCs from 43 AD patients and 41 normal subjects for fluorescence activated cell sorting (FACS), and some correlations were found among the Clinical Dementia Rating (CDR) score of AD patients, the numerical variation of B cells and the changes in the expression of marker genes as the disease developed. The indispensable function of B cells in AD progression was further validated using AD mice. We achieved proof-of-concept identification of possible pathogenic cell types and molecular biomarkers underlying AD, revealing the feasibility of identifying disease biomarkers in specific cell types rather than whole blood; the development of an AD diagnostic or early warning kit based on single-cell RNA-seq should be an urgent priority.

## Materials and methods

### Participants

Two normal individuals (Normal-1 (N1) and Normal-2 (N2)) and four AD patients (2 with mild AD, designated Early AD-1 (EA1) and Early AD-2 (EA2)), and 2 with severe AD, designated Late AD-1 (LA1) and Late AD-2 (LA2) were involved in single-cell sequencing analysis. Moreover, PBMCs from 43 AD patients and 41 normal subjects were collected for FACS and correlation analysis with the patients’ CDR scores. The patients fulfilled the following inclusion criteria^21^: a clinical diagnosis of mild or severe AD, a Mini-Mental State Examination (MMSE) score >19, an age range of 60–90 years, and stable administration of anti-dementia or mood-stabilizing medication. They were all informed of the purpose of the study and signed an informed consent form. Detailed information on these patients is shown in Supplementary Table 1. PBMCs were collected from patients and controls. All procedures involving the participants were approved by the Ethics Committee of Biomedical Research for West China Hospital (approval number 20150263; Chinese Clinical Trial Registry number ChiCTR1900022805), and this study was conducted in accordance with the Declaration of Helsinki.

### Magnetic resonance imaging

A 3.0 T magnetic resonance scanner (Ingenia, Philips, Netherlands) was used to detect cerebral structural changes in AD patients. The protocol and parameters for T1-weighted (T1W) imaging in all participants were as follows: repetition time (TR)/echo time (TE) = 500/20 milliseconds (ms), (number of signal averaged) NSA = 4, (field of view) FOV = 180 × 180 mm, matrix=224 × 224. The thickness of slices was set at 2 mm each.

### PBMC isolation

PBMCs in the blood samples obtained from normal individuals (N1 and N2) and AD patients (EA1 and EA2 for early AD; LA1 and LA2 for late AD) were purified by Ficoll gradient separation according to the manufacturer’s instructions (Ficoll-Paque Plus, GE Healthcare, Sweden). In short, 3 mL of Ficoll-Paque gradient was pipetted into two 15-mL centrifuge tubes. The blood was diluted at a ratio of 1:1 in phosphate-buffered saline (PBS) and carefully layered over the Ficoll-Paque gradient. The tubes were centrifuged for 30 min at 400 × *g*. The cell interface layer was harvested carefully (8 mL/tube), and the cells were washed twice in PBS for 10 min each (at 100 × *g*). PBMCs were resuspended in 1 mL complete culture medium (DMEM supplemented with 10% newborn calf serum and 1% PS). Cells were counted using a hemocytometer and trypan blue. PBMCs at a density of 3 × 10^6^ cells per tube were frozen in a liquid nitrogen tank.

### 10x genomics single-cell package and library preparation

Single-cell libraries were generated using a GemCode single-cell instrument and a 10x Genomics Single Cell 3′ Library & Gel Bead Kit v2 and Chip Kit according to the kit manufacturer’s protocol (Ou Yi Bio, Shanghai)^22^. Briefly, PBMCs were suspended in 0.04% BSA–PBS and then counted with a Countess® II Automated Cell Counter. The cell concentration was adjusted to the desired concentration of 1 × 10^6^/mL. Approximately 13000 cells were added to each channel. Nanoliter-scale gel beads in emulsion (GEMs) containing barcode information were generated and then reverse transcribed in a C1000 Touch Thermal Cycler (Bio-Rad). PCR amplification was performed using cDNA as a template. Subsequently, the amplified cDNA was fragmented into approximately 200–300 bp fragments by a Bioruptor sonication device, and the traditional second-generation sequencing process was followed, including, for example, sequencing linker P5 and sequencing primer R1. Finally, the DNA library was amplified by PCR.

### Single-cell RNA-seq

The library was quantified using Qubit, and the eligible libraries were placed in cBot for bridge amplification. All libraries prepared for this study were sequenced on a HiSeq 4000 system (Illumina) with 150 bp paired-end sequencing, and each library was sequenced for a whole lane. Briefly, fluorophore-labeled deoxyribonucleotide triphosphates (dNTPs) of A, T, G, and C were added during each cycle. According to AT and GC pairing, the corresponding dNTPs were bound to the template DNA strand by DNA polymerase, and the unbound dNTPs were washed out. After removal, the binding position released a fluorescent signal that could be captured by the computer and converted accordingly. Thereby, the base information for the binding position was obtained. The Cell Ranger software pipeline (version 2.2.0) provided by 10× Genomics was used to demultiplex cellular barcodes, map reads to the genome and transcriptome using the Spliced Transcripts Alignment to a Reference (STAR) aligner, and downsample reads as required to generate normalized aggregate data across samples, producing a matrix of gene counts versus cells. We processed the unique molecular identifier (UMI) count matrix using the R package Seurat^23^ (version 2.3.4). To remove low-quality cells and likely multiplet captures, which is a major concern in microdroplet-based experiments, we filtered out cells with UMI/gene numbers more than 4 standard deviations away from the mean value, assuming that the UMI/ gene numbers of the cells followed a Gaussian distribution. Following visual inspection of the distribution of cells by the fraction of mitochondrial genes expressed, we further discarded low-quality cells in which >10% of the counts belonged to mitochondrial genes. After the application of these quality control (QC) criteria, 46244 single cells (1304 cells filtered out from a total of 47548 cells) and 9279 genes remained and were included in downstream analyses. Library size normalization was performed in Seurat on the filtered matrix to obtain the normalized count.

### Data processing

The raw image data files obtained by single-cell RNA sequencing were converted into the original sequences by base-calling analysis. The resulting data were called raw data or raw reads. These results were stored in the FASTQ (.fq) file format, which contains the sequence information (reads) and its corresponding sequencing quality information. The top variable genes across single cells were identified using the method described by Macosko et al.^24^. Briefly, the average expression and dispersion were calculated for each gene, and the genes were subsequently divided into 18 bins based on expression. Principal component analysis (PCA) was performed to reduce the dimensionality of the log-transformed gene-barcode matrices of the top variable genes. Cells were clustered based on a gene-based clustering approach and visualized in 2 dimensions using t-distributed stochastic neighbor embedding (t-SNE) dimensionality clustering analysis (tsne.fit_transform(data_zs)). The cell type signature genes are listed in Table 1, the number of each cell subsets shown in Supplementary Table 2. A likelihood ratio test that simultaneously tests for changes in mean expression and in the percentage of expressed cells was used to identify significantly differentially expressed genes between clusters. The R package SingleR, a novel computational method for unbiased cell type recognition of single-cell RNA-seq, was used to infer the cell origin of each single cell independently and identify cell types by referring to the transcriptomic datasets “Blueprint Epigenomics”^25^ and “Encode”^26^. Differentially expressed genes (DEGs) were identified using the Find Markers function (test.use =MAST) in Seurat, and Pearson correlations between samples were calculated in R (cor(data, method = “pearson”)) based on the mean expression of the 693 highly variable genes. The criteria of *p* value < 0.05 and |log_2_(fold change)| > 0.26 were set as the thresholds for significantly differential expression. Heatmaps, volcano plots, and violin plots were generated using R. The enriched pathways associated with the differentially expressed genes were analyzed using the Kyoto Encyclopedia of Genes and Genomes (KEGG) database (https://www.kegg.jp/). Venn diagrams and circular heat maps were generated with TBtools (10.1016/j.molp.2020.06.009).

**Table 1:** The signature genes of 6 cell subsets.

Cell types	Signature genes
B cell	CD19, MS4A1
HSC	NCOR2, NKX3-1, HLX, PRNP
Monocyte	CLEC12A, MS4A6A, ZFP36L2
NK cell	GNLY, GZMB, SAMD3, DOCK2
CD4 + T cell	CD4, CD3D, CD3E, CD3G
CD8 + T cell	CD8A, CD8B, CD3D, CD3E, CD3G

### Fluorescence-activated cell sorting (FACS)

PBMCs from 43 AD patients and 41 normal subjects were isolated by FACS to further validate the results of single-cell sequencing. Briefly, all PBMCs were washed, counted and suspended in ice-cold PBS, and cell-surface antigen staining was subsequently performed. Monoclonal antibodies against CD3, CD45, CD56, CD19 (CD45/CD56/CD19/CD3 detection kit, Beckman Coulter, USA) and CD16 (CD16-PE, Beckman Coulter, USA) were used in immunofluorescence staining. The stained cells were analyzed and sorted by BD Influx (BD, USA), and data were analyzed using CytExpert 2.0.

### Animal care and grouping

Sixteen-week-old APP/PS1 transgenic mice (AD mice) and wild-type (WT) mice on a C57BL/6 genetic background were provided by the Center for Experimental Animals at Kunming Medical University. The animals were kept under standard conditions in a specific-pathogen-free (SPF) laboratory. All experimental procedures, including animal care and testing, conformed to the Animal Care and Use Committee of Kunming Medical University (kmmu2019058). All studies were conducted in accordance with the United States Public Health Service’s Policy on Humane Care and Use of Laboratory Animals. AD mice were randomly divided into an AD + B cell depletion treated (BCDT) group and an AD + PBS group. Mice in the AD + BCDT group received an intraperitoneal injection of 200 μL B cell depletion antibodies composed of 92 μL PBS, 55.5 μL CD19 and 52.5 μL B220 (anti-mouse CD19: 5.41 mg/mL, Bioxcell, Catalog #BE0150; anti-mouse B220: 5.71 mg/mL, Bioxcell, Catalog #BE0067) once every 5 days for 3 consecutive months, while the negative control mice received equivalent doses of PBS.

### Open field test

In this study, the open field test was mainly used to characterize the autonomous behavior, exploratory behavior and anxiety-like behavior of mice in the WT, AD control and AD treatment groups in an unfamiliar environment. The experimental setup consisted of two parts: the open field reaction box, which was 40 cm high with a 40 cm × 40 cm base and white interior walls, and the automatic data acquisition and processing system (Shanghai XinRuan Information Technology Co.). The enclosed part of the reaction box had a square floor area composed of 16 squares of 10 cm × 10 cm, with a digital camera positioned 2 m above the floor of the box. Each animal was placed in the center of the arena floor and had its behavior simultaneously videotaped and timed. Each mouse was observed for 10 min. The number of times the mouse reared and groomed as well as the cumulative time spent rearing and grooming was recorded. Between tests, the inner walls and floor of the box were cleaned.

### Morris water maze test

The Morris water maze was used to evaluate the spatial memory and learning abilities of AD mice. The pool, measuring 1.8 m in diameter and filled to a depth of 0.5 m, was divided into 4 equal quadrants, with a small round platform placed 1.5 cm below the surface of the water in the center of one quadrant. White food coloring was added to the water to hide the platform. The water maze experiment included a place navigation test and a spatial probe test. In the navigation test, the mice were placed at the midpoint of one randomly selected quadrant, and the time that the mice took to find the hidden platform (the latency) was recorded. For training, each mouse was released at the center of each of the 4 quadrants once per day for 5 days in a row; they were given up to 60 sec per trial to find the platform. If the mice failed to find the platform within 60 s during a training trial, they were guided to the platform by laboratory staff. On the sixth day, the platform was removed, and the spatial probe test began. The mice were placed in the water in the quadrant opposite the original location of the platform. The number of times each mouse crossed the target region, and the time and last day of training of distance that the mice traveled before reaching the target region were recorded and measured. Finally, the mice were dried after each experiment and returned to their home cages. The interval between training sessions was 15–20 min.

### Y-maze test

Mice were subjected to the Y-maze test at 3 months after BCDT injection. The Y maze consisted of three white plexiglass arms (each 35 cm long, 5 cm wide and 15 cm high) at an angle of 120° to each other. They were placed at the end of one arm and allowed to move through the maze. This test was divided into the following two parts. (1) *Spontaneous alternating experiment:* The order of entries into the three arms and the total number of entries into each arm were recorded. The alteration rate was calculated as the number of alterations/(total number entries-2) × 100. (2) *Spatial reference memory:* In the first training test, a certain arm (the novel arm) was closed, and the mice were allowed to explore freely for 15 min. Before the second test, the animals were returned to their cages for 1 h. During the second trial, the novel arm was made accessible, and the mice were allowed to move freely within the maze for 8 min. The number of times each mouse entered the novel arm and the total time spent in the novel arm were measured and analyzed.

### Tissue harvest

Twenty-four hours after behavioral experiments, all mice were anesthetized, killed, and immediately perfused with precooled 0.9% normal saline and 4% paraformaldehyde until the liver turned white and the body became stiff. Their brains were cut and fixed in 4% paraformaldehyde for 72 h, and trimmed blocks of brain tissue were dehydrated and embedded in paraffin. The region 2.66 mm~4.3 mm posterior to bregma was sectioned into coronal slices (5 μm thick), which were subsequently baked at 60 °C for 24 h and stored at room temperature for subsequent staining.

### Immunohistochemistry staining

The prepared brain sections were first deparaffinized and hydrated. After antigen retrieval with sodium citrate, these sections were washed 3 times with 0.01 M PBS for 5 min each, and then incubated with 3% hydrogen peroxide for 10 min to eliminate endogenous peroxidase. After three more washes with PBS, the sections were incubated with 5% goat serum and 0.3% Triton X-100 for 30 min at 37 °C. Afterward, primary antibodies (anti-β-amyloid 1–16, 6E10, Mouse, 1:1500) diluted in 2% goat serum were added and incubated for 18 h at 4 °C. Sections in the negative control group were treated with 2% goat serum. After being rinsed 3 times with PBS, they were incubated with an immunostaining chromogenic agent (MaxVision-HRP, mouse/rabbit) for 15 min at 37 °C and then developed by adding DAB solution. Finally, sections were dehydrated in gradient ethanol and xylene and sealed with neutral resins. The morphological changes in the brain tissues were observed under a light microscope. In five randomly selected fields, the Aβ plaque number and area and optical density value was calculated by Image Plus Pro.

### Statistical analysis

The sequencing data were analyzed using R software and Cell Ranger. All data are presented as the primary data or mean ± SEM. Statistical analysis was performed using SPSS 19.0 software. One-way analysis of variance (ANOVA) with Tukey’s post hoc test was applied for comparisons among multiple groups. The correlation among the number of T, B or NK cells; the expression levels of genes; and CDR scores (the severity of AD) was analyzed using the Pearson or Spearman correlation coefficient. The Pearson correlation coefficient was calculated based on the average expression of all cells in the corresponding group using R software (R: cor(data, method = “pearson”)) to visualize the intergroup variability across samples. A high correlation value represents high consistency in cell type distribution among samples, indicating low technical or biological variability across samples in the dataset^27^. The correlation between the patients’ age and the changes in the expression of differentially expressed genes in B cells was assessed using binary regression analysis. The genetic interaction was analyzed with String (https://www.string-db.org/), and interaction networks were generated by Cytoscape (Version 7.1). GraphPad Prism software version 7.0 (GraphPad Software Inc.) was used for quantification histogram generation. Any difference with a *p* value < 0.05 was considered significant.

## Results

### Analysis of the heterogeneity of PBMC populations by single-cell RNA-seq

**Fig. 1:**
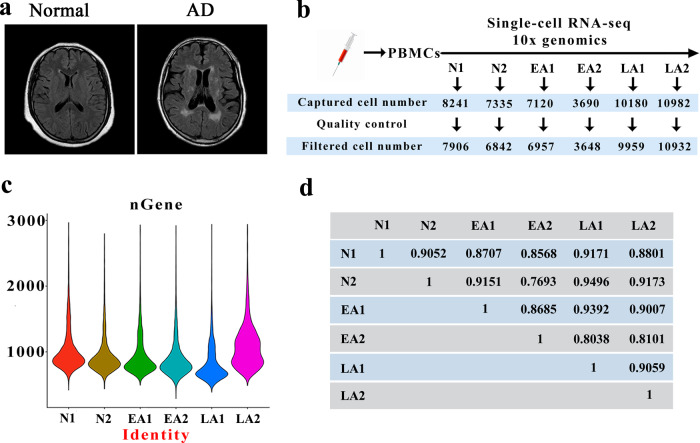
Heterogeneity of PBMC population analysis by single-cell RNA-seq. **a** MRI scanning images of the brains of a normal person and a representative late AD patient. **b** The number of single cells in each sample captured by single-cell RNA-seq 10X genomics. **c** The violin plot shows the gene number of each sample. **d** Pearson’s correlation plot visualizing the correlation (r) values between samples. N1, Normal-1; N2, Normal-2; EA1, Early AD-1; EA2, Early AD-2; LA1, Late AD-1; LA2, Late AD-2.

MRI scans showed cerebral atrophy and ventricular dilatation in late AD patients compared to normal individuals (Fig. 1a). PBMCs from AD patients were collected for single-cell RNA-seq based on 10x Genomics droplets. The numbers of captured cells in the 6 samples were 8241 for N1, 7335 for N2, 7120 for EA1, 3690 for EA2, 10180 for LA1 and 10982 for LA2, which decreased to 7906, 6842, 6957, 3648, 9959 and 10932, respectively, after quality control (Fig. 1b). Data from multiple sequencing runs were merged using the Cell Ranger pipeline. All cells were sorted according to the number of genes detected; a median of approximately 1000 genes were identified in these 6 samples, and more genes were identified in LA2 (a sample from a late AD patient) than in other samples (Fig. 1c). The data did not indicate that all cells in each sample had a total of 1,000 genes detected, and a median gene count of <1000 has been reported in previously published papers^28–30^. The intergroup variability in the datasets was visualized using the average expression of highly variable genes through Pearson correlation coefficients, which provided an overview of all variation between samples, with pairwise correlation values ranging from 0.8101 to 0.9496 (Fig. 1d). The correlation outcomes are shown in Supplementary Table 3, and the list of highly variable genes is available in Supplementary Table 4. Our datasets reveal high consistency in cell type distribution among samples.

### Verification of the differences in subsets of PBMCs in AD patients

**Fig. 2:**
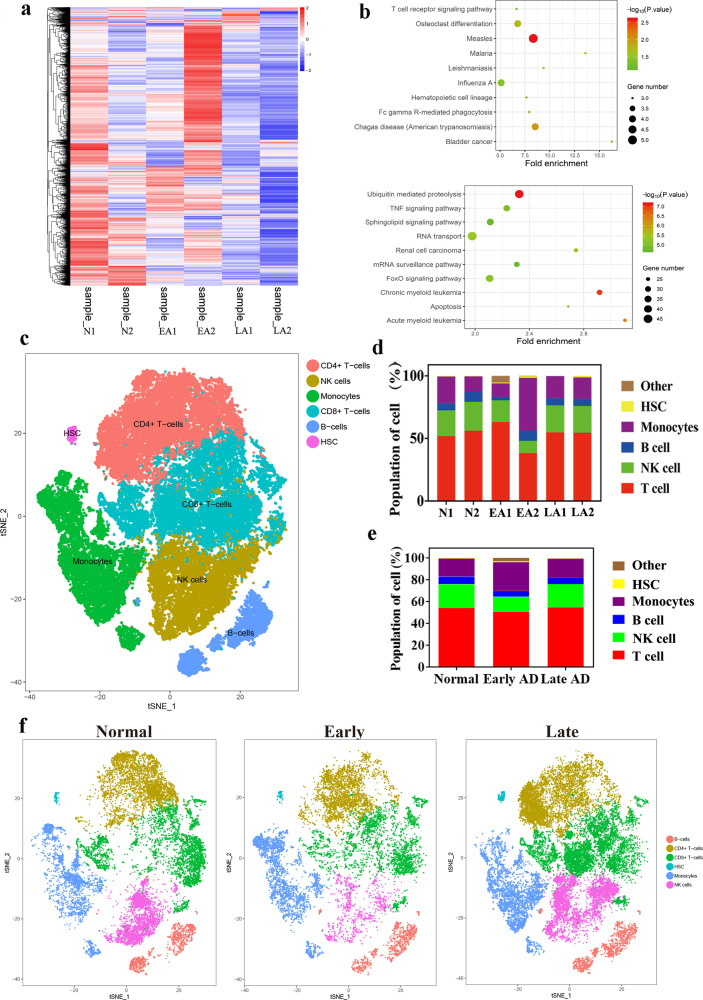
Verification of the differences in cell subsets of PBMCs in AD patients. **a** Heat map of differentially expressed genes in samples from AD patients and normal controls and (**b**) KEGG analysis of pathways involved in upregulated genes and downregulated genes. **c** t-SNE shows the top 6 cell subsets in all samples and (**d**) quantification of the proportion of cells in each sample. **e**, **f** The t-SNE map shows the different distribution of 6 clusters in normal, early AD, and late AD patients. The data were presented as the mean. The heatmap was generated by the pheatmap R package, and the rows were clustered. Pearson distance measurement assigns values to the clustering rows. The bubble diagram was generated by the ggplot R package. The color of the bubbles varies from red to green. The redder the bubbles are, the greater the -log_10_(*p* value), that is, the smaller the *p* value. The larger the bubble is, the more genes are enriched. N1, Normal-1; N2, Normal-2; EA1, Early AD-1; EA2, Early AD-2; LA1, Late AD-1; LA2, Late AD-2.

KEGG analysis of differentially expressed genes in 6 cell subsets revealed that the upregulated genes identified by single-cell RNA-seq were mainly enriched in the pathways Measles, Influenza A, Chagas disease (American trypanosomiasis), Bladder cancer, Malaria, Leishmaniasis, Fc gamma R-mediated phagocytosis, Hematopoietic cell lineage, Osteoblast differentiation and T cell receptor signaling pathway, while the downregulated ones showed enrichment in Ubiquitin-mediated proteolysis, Chronic myeloid leukemia, Acute myeloid leukemia, Renal cell carcinoma, RNA transport, FoxO signaling pathway, TNF signaling pathway, Apoptosis, Sphingolipid signaling pathway and mRNA surveillance pathway (Fig. 2a, b). Through the gene-based cluster analysis, the top 6 cell subsets in these 6 samples were screened and overlaid in 2 dimensions (Fig. 2c), and their proportion in each sample was displayed (Fig. 2d, Supplementary Table 2). Collectively, the quantification data showed high densities of T cells and NK cells in each sample (Fig. 2d, Supplementary Table 2). In the PBMCs of early and late AD patients, the percentage of B cells was visibly decreased compared with that of normal controls (Fig. 2e, f, Supplementary Table 2).

### Gene expression profiles of cell subpopulations of PBMCs in AD patients

**Fig. 3:**
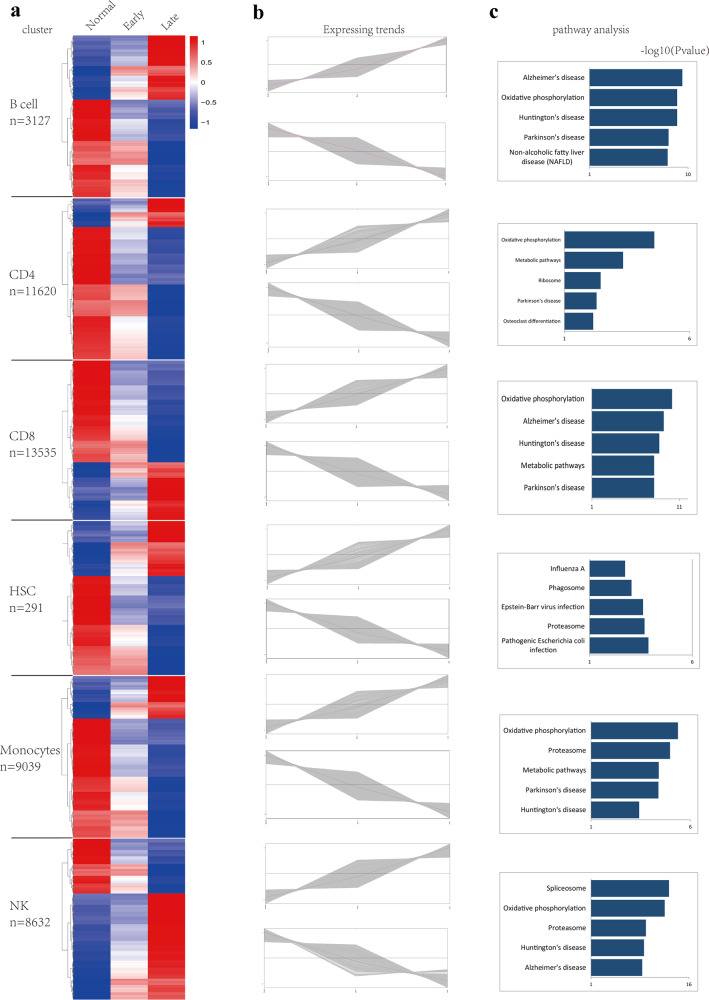
Gene expression profiles of cell subpopulations of PBMCs in AD patients. **a** Heat map of changes in the RNA expression of upregulated and downregulated genes in the top 6 cell types in normal people and early and late AD patients. N values below the cell names represent the cell counting results. **b** The gene expression changes trends of upregulated and downregulated genes in 6 different cell types. **c** KEGG pathway analysis of upregulated genes in 6 cell types. The heatmap was generated by the pheatmap R package, and the rows were clustered. Pearson distance measurement assigns values to the clustering rows. The line chart was generated by the ggplot R package. The bar chart of KEGG analysis was made by the hist function of R language in accordance with the -log_10_ (*p* value).

We performed clustering analysis to examine cellular heterogeneity in each subset of PBMCs. To identify cluster-specific genes, the difference in the expression of each gene at different stages of AD was calculated. The heatmap demonstrated the difference in the expression of the significantly upregulated and downregulated genes in single-cell gene expression data of the 6 cluster-specific cells (B cells, CD4, CD8, HSCs, monocytes and NK cells) of AD patients and normal individuals (Fig. 3a). As shown, differentially expressed genes in each cell subset was divided into upregulated and downregulated genes according to the trends in their expression as the disease proceeded (Fig. 3a, b). KEGG analysis of differentially expressed genes in each cell subtype was carried out, revealing the top five pathways in which the upregulated genes of each cell type was enriched. The pathways were as follows. *B cells:* Alzheimer’s disease, Oxidative phosphorylation, Huntington’s disease, Parkinson’s disease, Nonalcoholic fatty liver disease (NAFLD); CD4+ *T cell:* Oxidative phosphorylation, Metabolic pathways, Ribosome, Parkinson’s disease, Osteoclast differentiation; CD8+ *T cell:* Oxidative phosphorylation, Alzheimer’s disease, Huntington’s disease, Metabolic pathways, Parkinson’s disease; *HSCs*: Pathogenic *Escherichia coli* infection, Proteasome, Epstein-Barr virus infection, Phagosome, Influenza A; *Monocytes:* Oxidative phosphorylation, Proteasome, Metabolic pathways, Parkinson’s disease, Huntington’s disease; *NK cell:* Spliceosome, Oxidative phosphorylation, Proteasome, Huntington’s disease, Alzheimer’s disease (Fig. 3c). The pathways in which downregulated genes were enriched were almost entirely consistent with the results shown in Fig. 2a. In summary, upregulated genes in B cells, CD8+ T cells and NK cells all exhibited enrichment in Alzheimer’s disease, which took first place in B cells, second in CD8+ T cells and fifth in NK cells among the top five enriched signaling pathways.

### Differential gene expression profiles in B cells

**Fig. 4:**
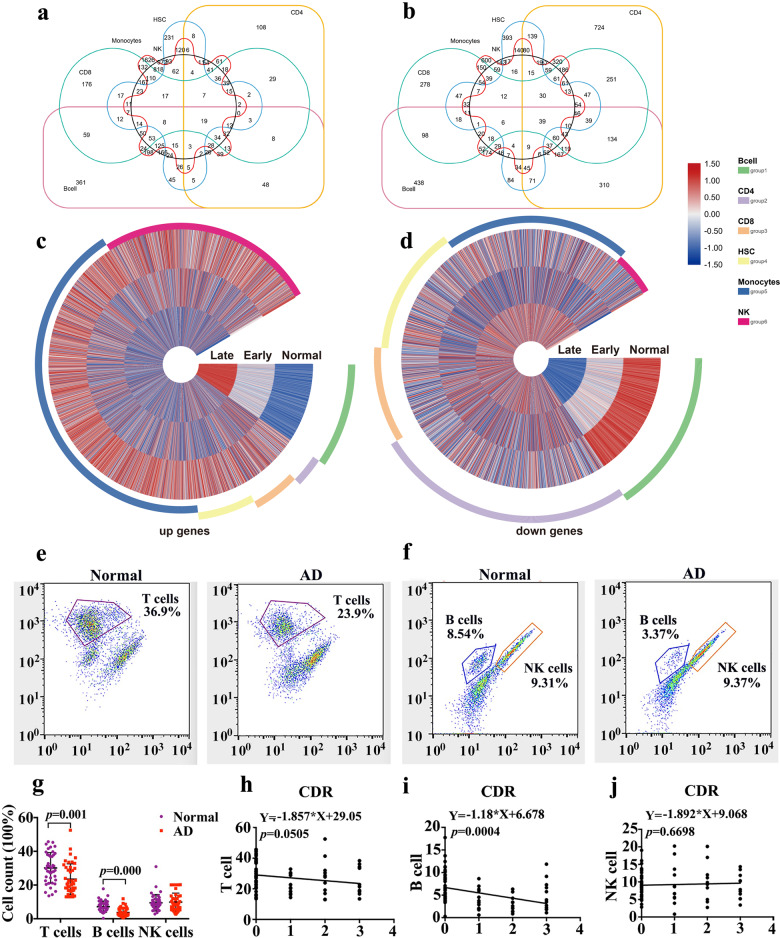
Differential gene expression profiles in B cells. Venn diagram of (**a**) upregulated and (**b**) downregulated genes in different cell types. The specifically (**c**) upregulated and (**d**) downregulated genes in B cells and significant alterations in B cells revealed by the circular heat map. **e**, **f** PBMCs of 43 AD patients and 41 normal subjects were harvested and subjected to flow cytometry with a panel of T cell, B cell and NK cell marker monoclonal antibodies. **g** The positive cell numbers of T cells, B cells and NK cells (mean ± SEM). **h**–**j** Correlation analysis of CDR with the number of T cells, B cells and NK cells. The Venn diagram and Circosheatmap were generated by TBtool.

A Venn diagram made with TBtools (10.1016/j.molp.2020.06.009) revealed the numbers of specific upregulated and downregulated genes in the 6 different cell types. Excluding the overlapping areas, there were 361 specific upregulated genes and 438 specific downregulated genes found in B cells, 108 upregulated and 724 downregulated genes in CD4 + T cells, and 176 upregulated and 278 downregulated genes in CD8 + T cells (Fig. 4a, b). A circular heat map made with TBtools (10.1016/j.molp.2020.06.009) displayed how the genes that were specifically expressed in B cells were expressed in the other 5 cell types, and the results revealed that the genes that were specifically upregulated or downregulated in B cells did not exhibit obvious changes in other cell subsets (Fig. 4c, d). In addition, according to inclusion criteria for AD, PBMCs from 43 AD patients and 41 normal subjects were collected for FACS, we found that AD patients had fewer T cells (23.9%) and B cells (3.37%) than normal controls (36.9% and 8.54%, respectively; Fig. 4e, f, g; *p* = 0.000 and *p* = 0.001, respectively). The relative number of NK cells was not significantly different. Furthermore, the correlation analysis between the number of B cells and CDR score showed that the number of B cells was negatively related to the severity of AD; that is, the fewer B cells there were, the more serious the development of AD was (Fig. 4h, i, j, *p* = 0.0004). Based on these findings, B cells were determined to be a specific biomarker for AD.

### B cell depletion causes cognitive decline in AD mice

**Fig. 5:**
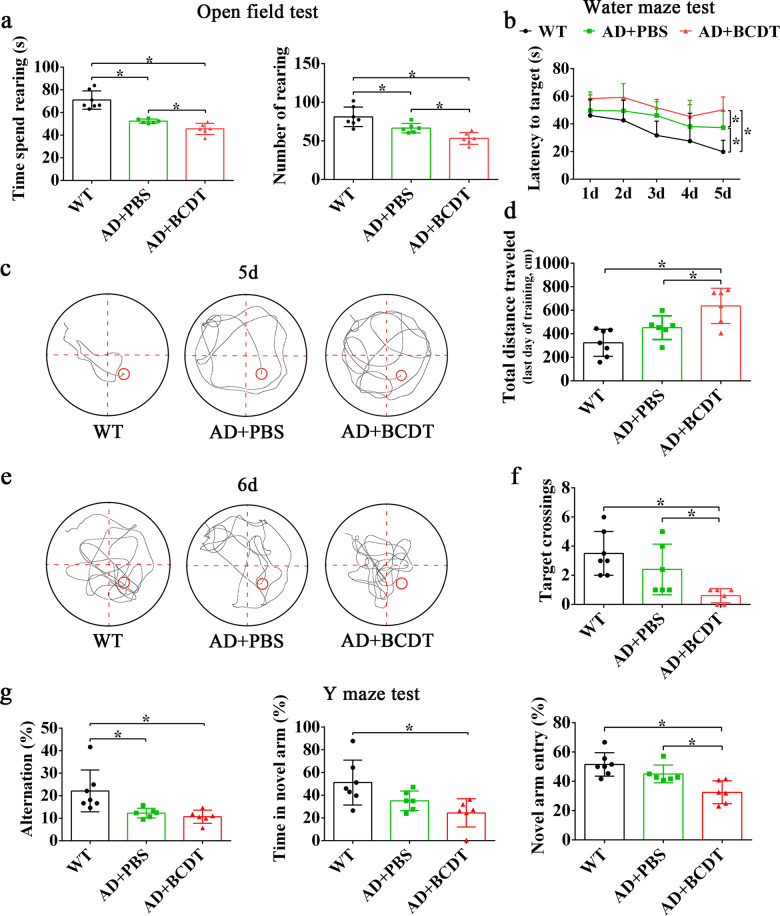
Functional verification of B cells in AD mice. **a** The time spent and the number of rearing in the mice from the WT, AD + PBS and AD + BCDT groups. **b** The latency to target, **c**, **e** the motion trail at 5th day and 6th day of mice from WT, AD + PBS and AD + BCDT groups in Morris water maze. **d** The distance traveled and (**f**) the number of target crossings in mice of three groups. **g** The alteration rate, the time spent in the novel arm and the novel arm entry rate of mice in the three groups in the Y-maze. WT wild-type (mice), BCDT B cell depletion treated.

To further verify the function of B cells in the progression of AD, 16-week-old AD mice were subjected to B cell depletion. After three months of consecutive BCDT, cognitive function was evaluated by the open field test, Morris water maze test and Y-maze test. As shown, the AD mice treated with PBS and BCDT performed worse, as indicated by decreased rearing duration and number of rearing events, than the dementia-free WT mice (Fig. 5a, *p* < 0.05), and the mice in the AD + BCDT group suffered more severe dysfunction than those in the AD + PBS group (*p* < 0.05). The mice in the AD + BCDT and AD + PBS groups also performed worse in the Morris water maze and Y-maze tests than WT controls. A prolonged escape latency time, an increased distance traveled to the platform, and a reduced number of platform area crossings demonstrated that AD mice had significant spatial and learning disabilities compared to controls, and the disabilities in the AD + BCDT group were much more severe than those in the AD + PBS group (Fig. 5b, c, d, e, f, *p* < 0.05). The decreased working memory abilities of AD mice were indicated by shorter duration in the novel arm, fewer entries into the novel arm, and lower spontaneous alteration than control mice in the Y-maze test (Fig. 5g, *p* < 0.05).

### B cell depletion increased Aβ deposits in the brains of AD mice

**Fig. 6:**
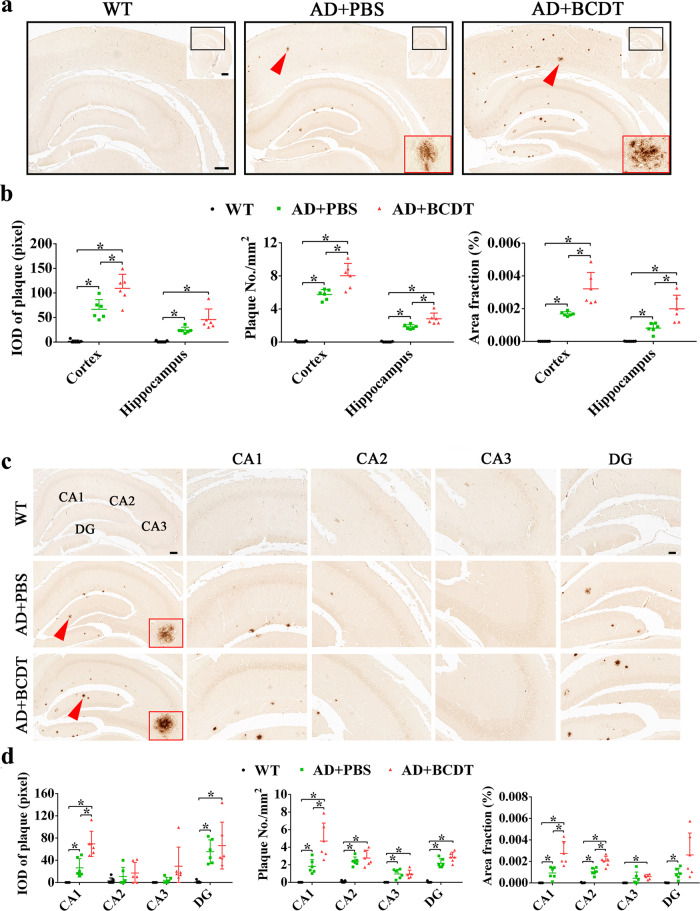
The effect of B cell depletion on Aβ deposition. **a** Histological images of Aβ plaque distribution in the brain among the WT, AD + PBS and AD + BCDT groups. Scale bar = 20 μm and 50 μm. **b** Quantification data including integrated optical density (IOD) of Aβ plaque, the number of plaques and the area of plaques in WT, AD + PBS and AD + BCDT groups. **c** The distribution of Aβ plaques in the hippocampus (CA1, CA2, CA3, DG) among the three groups. Scale bar = 100 μm and 400 μm. **d** The IOD, amount and area of Aβ plaques in the four regions of the hippocampus among the three groups. WT, wild-type (mice); BCDT, B cell depletion treated.

The immunohistochemistry results showed that the amyloid plaque burden in the hippocampus and cortex were increased in PBS and BCDT AD mice compared to WT mice (Fig. 6a, *p* < 0.05). The optical density value of plaques, the number of plaques and the total plaque area in the cortex and hippocampus were all significantly elevated in the AD mice relative to the WT mice (*p* < 0.05), and those of AD mice with BCDT were increased more than those of AD mice treated with PBS (Fig. 6b, *p* < 0.05). The distribution of Aβ plaques in the four regions of the hippocampus was further specified. The plaques were mainly distributed in the CA1 region and DG of the hippocampus in the AD mice (Fig. 6c), as indicated by the increased OD value of plaques in those regions among AD mice compared to WT mice (*p* < 0.05). The BCDT AD mice exhibited higher plaque OD values in the CA1 and DG than PBS-treated AD mice (Fig. 6d, *p* < 0.05). Meanwhile, the number of plaques and the ratio of plaque area to total hippocampus area were elevated in all four regions of hippocampus in the AD mice compared to WT mice, and those in BCDT AD mice were even higher than those in PBS-treated controls (Fig. 6d, *p* < 0.05).

### Identification of specific differential gene expression in B cells

**Fig. 7:**
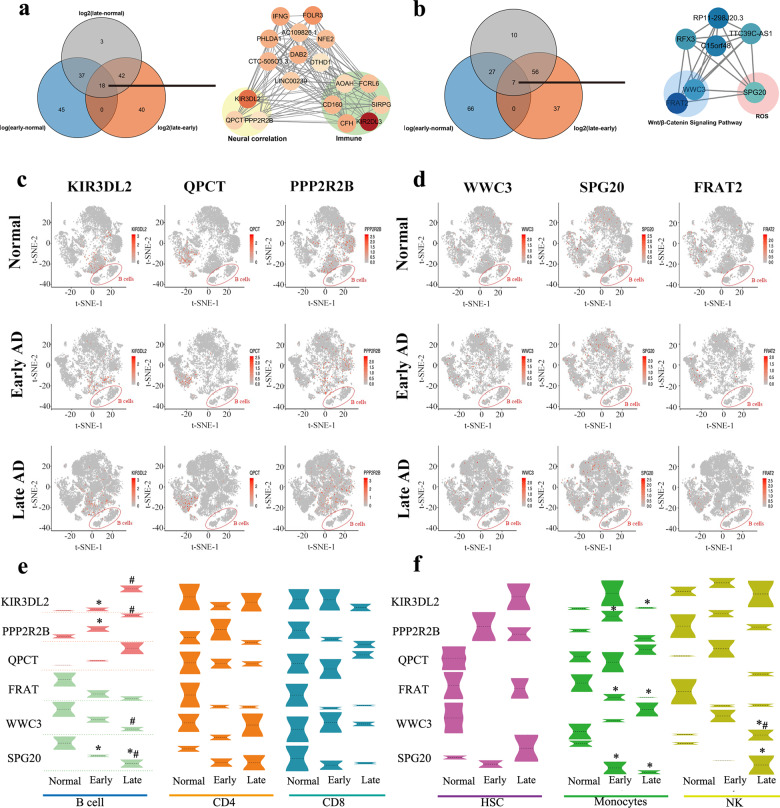
The identification and verification of specific genes. **a** Venn diagram of the top 100 upregulated genes with the highest fold change among normal people and patients at different stages of AD (left) and interactive relationships among the 18 overlapping genes by Cytoscape (right). The darker the red color is, the greater the fold change in expression in the late-stage group, and the more significant the upregulation of genes. **b** Venn diagram of the top 100 downregulated genes with the highest fold change among normal people and patients at different stages of AD (left) and interactive relationship among the 7 overlapping genes by Cytoscape (right). The darker the blue color is, the greater the fold change in expression in the late-stage group, and the more significant the downregulation of genes. **c** The t-SNE map shows the expression variation of three specifically upregulated genes in B cells at normal, early, and late stages of AD. **d** The t-SNE map shows the expression variation of three specifically downregulated genes in B cells at normal, early, and late stages of AD (mean ± SEM). **e**, **f** Violin plots showing the expression variation of KIR3DL2, PPP2R2B, QPCT, FRAT2, WWC3 and SPG20 in 6 types of cells (B cells, CD4, CD8, HSCs, monocytes and NK cells) in normal controls and patients with early and late stages of AD. *p < 0.05 vs. normal group, ^#^*p* < 0.05 vs. early AD group.

The top 100 upregulated genes (ranked by fold change) expressed in B cells during the progression of AD were identified, and the overlapping area showed 18 prominent genes as the disease proceeded: AC109826.1, LINC00239, CTC-505O3.3, DTHD1, CFH, NFE2, QPCT, DAB2, PHLDA1, PPP2R2B, SIRPG, KIR2DL3, FOLR3, KIR3DL2, AOAH, IFNG, CD160, and FCRL6. These factors were then imported into String, and their correlation and interaction were revealed and displayed by Cytoscape (Fig. 7a). Likewise, the overlapping downregulated genes were also determined and imported into String to elucidate their interactive relationships; these genes were TTC39C-AS1, FRAT2, WWC3, C15orf48, RFX3, RP11-298J20.3, and SPG20. In B cells, the upregulated genes KIR3DL2, QPCT, and PPP2R2B are all involved in neural function and neurodegenerative diseases, and the downregulated genes FRAT2 and WWC3 are involved in the Wnt/β-Catenin signaling pathway, which is associated with AD pathogenesis^31,32^ (Fig. 7b). Downregulated SPG20 could mediate mitochondrial calcium homeostasis. Dysfunction in regulating mitochondrial calcium homeostasis can bring about intracellular accumulation of reactive oxygen species (ROS), resulting in oxidative stress, which is one of the early features of AD. Thus, these 6 genes were selected for further focus. We further analyzed the aforementioned differentially expressed genes in B cells from single-cell RNA-seq data; we found that KIR3DL2, QPCT and PPP2R2B were increasingly expressed above the normal baseline in B cells as the disease progressed, while the expression of SPG20, FRAT2 and WWC3 decreased from the normal baseline as the disease developed (Fig. 7c, d).

### Expression of specific genes in each cell subset with the progression of AD

To verify the specific expression trends of the abovementioned upregulated and downregulated genes in B cells, we analyzed the expression trends of these 6 genes in B cells together with those in other cell clusters (CD4, CD8, HSC, monocytes, NK cells) from the single-cell RNA-seq data. As shown, the expression of KIR3DL2 and PPP2R2B was significantly upregulated in B cells in early and late stages of AD (Fig. 7e, *p* < 0.05), while the same was not true in other cell clusters. WWC3 and SPG20 exhibited marked downregulation in B cells as AD proceeded (Fig. 7e, *p* < 0.05). Additionally, in monocytes, the levels of PPP2R2B were significantly elevated while those of SPG20 showed an obvious decrease during the progression of AD (Fig. 7f, *p* < 0.05). Depending on whether the changes in the expression of these 6 factors deviated from the values in normal patients, the expression data were divided into those with normal tendencies and those with abnormal tendencies, and the patients’ age was included for binary regression analysis. The results showed that age was not a risk factor for genetic change (*p* = 0.994).

## Discussion

In this study, differentially expressed cell subpopulations were screened in PBMCs of AD patients by single-cell sequencing analysis; B cells represented a crucial cell subset in the progression of AD, as verified through in vivo functional experiments in AD mice. In addition, differentially expressed genes in B cells were identified; both the upregulated genes (KIR3DL2, QPCT, PPP2R2B) and the downregulated genes (FRAT2, WWC3, SPG20) had certain connections with neurodegenerative diseases. In the late stage of AD, KIR3DL2, QPCT and PPP2R2B in B cells were expressed at considerably higher levels than in early AD, while FRAT2, WWC3 and SPG20 showed the opposite expression pattern.

The study of biomarkers for the diagnosis of AD has been a challenging undertaking. Morphophysiological characterization with slice physiology has been regarded as the standard in AD prognosis for decades^33^, but this method has many problems, such as insufficient sampling, difficulty in quantitative classification of cell types and limited extensibility in terms of neuron diversity^33,34^. Single-cell RNA-seq allows unbiased, high-throughput quantitative investigations of molecularly defined cell types in any species, including human beings^35,36^. However, its scalability has been limited by throughput (a maximum of 96 cells per microfluidic chip), high cost, and sampling bias arising from poor capture of smaller nonneuronal nuclei on microfluidic chips^37^. Transcriptome analysis can identify different functional cell types making up complex tissues, and the inclusion of epigenetic information can provide a more complete picture of how these expression profiles are regulated or maintained. Such recognition of cell-type-specific regulation will help improve the understanding of the genetic programs that define the processes of cell commitment and differentiation^38^. In addition, since the common genetic variants associated with different traits and diseases are mainly located in intronic or intergenic regions and enriched in tissue-specific regulatory sites^39^, the generation of cell-specific regulome maps may provide additional valuable insights into the important mechanisms underlying diseases^40^. Single-cell RNA-seq has been used to classify cells in the spleen, lung epithelium, and embryonic brain^41^. Thus, higher-throughput single-cell RNA-seq approaches specifically applicable to humans are necessary.

Increasing evidence demonstrates the therapeutic relevance of PBMCs to AD^13^. Immune cells, particularly lymphocytes, may be involved in the pathogenesis of AD^42^. Hence, elucidation of specific cellular and gene expression changes may be helpful in the early diagnosis of AD. To understand cellular heterogeneity and identify key genes, appropriate experimental and computational methods are needed to make full use of the application of single-cell RNA-seq. In this study, single-cell RNA-seq was employed to analyze differential cells and genes in PBMCs of AD patients. The single-cell RNA-seq results in this work provide a detailed view of PBMCs in normal and AD patients, in which the top 6 cell subsets were B cells, CD4 + T cells, CD8 + T cells, HSCs, monocytes and NK cells, and the cell difference in each cluster can be clearly seen. Furthermore, while studying signaling pathways, KEGG analysis revealed that the upregulated genes in the PBMCs were mainly enriched in the pathways Measles, Influenza A, Chagas disease (American trypanosomiasis), Bladder cancer, Malaria, Leishmaniasis, Fc gamma R-mediated phagocytosis, Hematopoietic cell lineage, Osteoblast differentiation and T cell receptor signaling pathway, while the downregulated genes in the PBMCs showed enrichment in the pathways Ubiquitin-mediated proteolysis, Chronic myeloid leukemia, Acute myeloid leukemia, Renal cell carcinoma, RNA transport, FoxO signaling pathway, TNF signaling pathway, Apoptosis, Sphingolipid signaling pathway and mRNA surveillance pathway, all of which were independent of Alzheimer’s disease. However, when the gene expression of each cell subpopulation was targeted, Alzheimer’s disease enrichment ranked first among the signaling pathways enriched for upregulated genes in B cells. In addition, upregulated genes in CD8 + T cells and NK cells were also enriched in Alzheimer’s disease, ranking second and fifth, respectively. Immediately, the investigation value of B cells in AD stands out of other cell subsets. Afterward, the results were continuously expanded in 43 AD patients and 41 normal subjects through FACS, B cells were found to be specifically reduced in PBMCs of AD patients after the initial analysis, and the decreased number of B cells was closely correlated with AD severity. Similar findings were shown in a previous study, in which the absolute number and percentage of B cells in the lymphocyte subsets of AD were depressed relative to normal controls, whereas the other subsets displayed no significant difference^43–45^, which further confirmed the reliability of our single-cell RNA-seq results. To verify the role of B cells in AD progression, we investigated their function by depleting B cells from 16-week-old APP/PS1 mice. After 3 months (once every 5 days) of intraperitoneal injection of anti-CD19/B220 antibodies, neurobehavioral tests were performed at the 28^th^ week, and immunohistochemical staining was performed at the 30^th^ week, which revealed that AD mice with B cell inactivation suffered much more severe cognitive deficits than AD controls. Correspondingly, the brains of B cells removed exhibited a significantly increased number and area of Aβ plaques in the cortex and hippocampus. Specifically, Aβ plaques were mainly distributed in the CA1 region and DG of the hippocampus of AD mice. Functionally, the rostral and caudal hippocampus is known to be involved in different forms of learning and memory^46^. Consistent with our observation, one MRI study of 120 participants with AD demonstrated that hippocampal subregions underwent differential atrophy^47^.

In our data, inactivation of B cells in the early stage significantly aggravated the AD-induced cognitive barriers with an elevated number and area of Aβ plaques in AD mice. Recently, B cell–related processes in AD have been the topic of many investigations, some of which consistently revealed that B cells produce immunoglobulins that are potentially beneficial for reducing Aβ plaques^48^ and that they express AD-ameliorating cytokines^49^. Nevertheless, a “dark” side of B cells was reported, as they exacerbated manifestations of AD-like symptoms. B cells in AD mice appeared to lose their anti-inflammatory activity and took on an inflamed phenotype, indicated by upregulation of proinflammatory cytokines^50^ expression and B cell colocalization with Aβ plaques and activated microglia. B cells together with T cells constitute the adaptive immune system, and B cells have capabilities of fulfilling various cellular and humoral functions that are dependent on their stage of differentiation and activation status^51^. There is no lack of conflicting outcomes reporting the pathogenic or protective roles of B cells in AD mice. In a recent study by Kim et al., the loss of B cells benefited AD patients by reducing Aβ plaque burden and disease-associated microglia, reversing behavioral and memory deficits and restoring TGFβ + microglia^50^. To clarify the causes of the disagreement between our results and theirs, we carried out a detailed comparison of the murine experiments between the two articles and summarized the differences. It is noted in a published study that B cell deficiency transgenic mice were constructed in 3×TgAD and APP/PS1 mice by crossing 3×TgAD or APP/PS1 mice with J_H_T mice, and transient B cell inactivation or depletion was generated in 60- to 70-week-old 3×TgAD and 35- to 47-week-old 5×FAD mice by intraperitoneal injection of anti-B cell antibodies, anti-CD20 (100 μg/mouse) and anti-B220 (250 μg/mouse). There was no overlap with regard to the arrangement of B cell depletion modeling, as in our study, 16-week-old APP/PS1 mice received intraperitoneal injection of 200 μL anti-CD19 (300 μg/mouse) and anti-B220 (300 μg/mouse) for B cell clearance. APP/PS1 mice are known as a classic model of early onset of AD and exhibit earlier AD pathology than 3×TgAD mice. B cell depletion in APP/PS1 mice was initiated in our study when fibrous plaques were observed in mice at 16 weeks of age (data not shown), which was younger than the age at which B cell APP/PS1 knockout mice were used (20–35 weeks). In addition, in their operation on another two models, B cells were cleared from 3×TgAD (60–70 weeks) and 5×FAD (35–47 weeks) mice. In view of the frequency and duration of injection, we completed 21 injections within 3.5 months, including two weeks for behavioral tests to eliminate B cells, compared to 3–6 injections within 2 months in their research, and our design maintained consecutively adequate depletion of B cells in the early onset of AD. Importantly, several factors are needed to determine the pathogenic or protective roles of B cells, including the source of B cells (brain, cerebrospinal fluid or blood), antigen specificity of B cell clonotypes, B cell phenotyping^51^ and so on. Hence, the aforementioned discrepancies may lie in the different B cell depletion antibodies, injection frequency of antibodies, injection duration, strain and age of animals. In summary, despite their counterintuitive finding that B cell depletion had a protective effect in AD, our study proposes that massive B cell depletion in the early onset would exacerbate AD progression, which is in concordance with the double-edged role of B cells in neurodegenerative disorders^51^. In this study, we base our outcomes on single-cell RNA-seq data of human PBMCs and functional experiments on transgenic AD mice. The existing insufficient evidence for the protective effects of B cells in the early onset of AD warrants further in-depth clinical investigations to facilitate the development of novel therapies for AD treatment.

It is possible that the alteration of the peripheral B cell compartment in certain neurodegenerative diseases, such as AD, is attributed to genetic factors. Consistent with the present study, single-cell RNA-seq data further uncovered that among 18 specifically upregulated genes, the expression of KIR3DL2, PPP2R2B and QPCT in B cells was correlated with neural function and neurodegenerative diseases^52–54^. Of 7 specifically downregulated genes, FRAT2 and WWC3 were involved in the Wnt/β-Catenin signaling pathway, which was associated with AD pathogenesis^55,56^. SPG20 is capable of regulating mitochondrial calcium homeostasis^57^, indirectly associated with AD, because intracellular accumulation of ROS would occur as a result of dysfunction in regulating mitochondrial calcium homeostasis, leading to oxidative stress, which is one of the early features of AD^58^. Additional evidence in this study verified the specificity of these upregulated and downregulated genes in B cells instead of in other cell subsets via t-SNE map analysis. These results implied that the upregulation of KIR3DL2, PPP2R2B and QPCT and the downregulation of FRAT2, WWC3 and SPG20 in peripheral B cells can serve as a gene panel of biomarkers for the diagnosis and prognosis of AD.

Collectively, both differential cell subpopulations in PBMCs and specifically expressed genes in B cells of AD patients were identified in this work using single-cell RNA-seq analysis. A reduction in B cells in the PBMCs of AD patients and differentially expressed genes in B cells were identified in the present study, indicating that certain important genes that could be molecular biomarkers in AD development may be expressed in certain cell types. The importance of promising single-cell RNA-seq analysis in AD biomarker investigations has been strengthened. This study in its present form merely identified possible biomarkers of AD progression. Limitations exist because the obtained specific genes lack longitudinal investigations to further determine their specific roles in the development of AD, which will be worked out in later clinical and basic research.

## Supplementary information

Supplemental materials

## Data Availability

The RNA-seq data reported in this article have been deposited in the National Center for Biotechnology Information (NCBI) Gene Expression Omnibus (GEO) and are accessible through GEO Series accession number GSE168522. The detailed analysis method used in this study is provided in the Methods section and listed in Supplementary Table 5.
