# Supplementary material for: Single-cell RNA sequencing reveals B cell–related molecular biomarkers for Alzheimer’s disease
Source: Exp Mol Med. 2021 Dec 8;53(12):1888–901. doi: 10.1038/s12276-021-00714-8 (PMC8741783; doi:10.1038/s12276-021-00714-8)
Supplement: Supplementary file 1 — Supplemental materials [file 12276_2021_714_MOESM1_ESM.pdf]

Supplementary Table 1 The basic information of the participants involved in this study

Group	Sample ID	Gender	Age	Profession	Education	Electroencephalogram (EEG)
Normal-1	16	Female	67	Retired	Secondary technical school	Normal
Normal-2	17	Female	73	Retired	Secondary technical school	Normal
Early AD-1	23	Female	71	Retired	Middle school	Mildly to moderately abnormal
Early AD-2	28	Female	73	Retired	Middle school	Mildly to moderately abnormal
Late AD-1	24	Female	82	Retired	Junior college	Severely abnormal
Late AD-2	27	Male	90	Retired	Middle school	Moderately to severely abnormal

Supplementary Table 2 The number of 6 cell subsets in PBMCs

Cell number of each subsets						
Sample	N1	N2	EA1	EA2	LA1	LA2
B cell	451	578	189	296	578	638
NK cell	1621	1569	1201	355	2144	2322
T cell	4092	3842	4390	1398	5454	5967
Monocytes	1691	817	753	1535	1761	1858
HSC	44	32	38	46	14	123
Other	7	4	386	18	8	24
Total	15761	13648	13490	7232	19896	21717

The number of 6 cell subsets in PBMCs

Ratio of each subsets						
Sample	N1	N2	EA1	EA2	LA1	LA2
B cell	2.8614936	4.2350528	1.4010378	4.0929204	2.9051066	2.9377907
NK cell	10.28488	11.49619	8.902891	4.9087389	10.776035	10.692085
T cell	25.96282	28.150645	32.542624	19.330752	27.412545	27.476171
Monocytes	10.729015	5.9862251	5.5819125	21.225111	8.8510253	8.5555095
HSC	0.2791701	0.2344666	0.2816901	0.6360619	0.0703659	0.5663766
Other	0.0444134	0.0293083	2.8613788	0.2488938	0.0402091	0.1105125

Supplementary Table 3 Correlation of highly variable genes

	N1	N2	EA1	EA2	LA1	LA2
N1	1	0.90518318	0.870696769	0.856768532	0.917056107	0.880107255
N2	0.90518318	1	0.915115063	0.769323643	0.949631869	0.917261986
EA1	0.870696769	0.915115063	1	0.868476227	0.939178696	0.900686088
EA2	0.856768532	0.769323643	0.868476227	1	0.803819156	0.810059295
LA1	0.917056107	0.949631869	0.939178696	0.803819156	1	0.905919497
LA2	0.880107255	0.917261986	0.900686088	0.810059295	0.905919497	1

Supplementary Table 4 List of highly variable genes

HES4	ISG15	TNFRSF18	TNFRSF4	RP5-832 C2.5	MMP23B	RBP7	CDA	C1QA	STMN1

ZNF683	IFI6	CCDC28B	MARCKSL1	CSF3R	MOB3C	PDZK1IP1	RNF11	JUN	GADD45A
NEXN	RP11-386I14.4	IFI44L	RP5-887A10.1	LMO4	TGFBR3	GFI1	GCLM	CHI3L2	RHOC
HIPK1-AS1	TSPAN2	CH17-373J23.1	CD160	HIST2H2BE	CTSS	C1orf56	S100A12	S100A8	LMNA
SH2D2A	FCRL3	FCRL1	CD1C	MNDA	FCER1A	FCRL6	SLAMF7	CD244	FCER1G
FCGR3A	FCRLA	SH2D1B	UAP1	CD247	XCL2	XCL1	FASLG	RALGPS2	IER5
NCF2	C1orf21	PTGS2	RGS18	RGS1	RGS2	G0S2	LYPLAL1	CNST	AHCTF1
RSAD2	AC092580.4	ID2	ODC1	RHOB	CYP1B1	BCL11A	CAPG	CD8A	CD8B
AC133644.2	RP11-1399P15.1	IGKC	MAL	DUSP2	IL18RAP	LIMS1	IL1B	MARCO	PTPN18
CXCR4	HNMT	NR4A2	CD302	GCA	PDK1	CHN1	SLC40A1	C2orf88	SDPR
AC079767.4	TUBA4A	ITM2C	NMUR1	RAMP1	HES6	PASK	BHLHE40	TMEM40	ANKRD28
RP11-222K16.2	EOMES	CMC1	CTDSPL	CX3CR1	GPX1	FHIT	FRMD4B	PROK2	NFKBIZ
TIGIT	CSTA	MGLL	GP9	H1FX	TFDP2	GK5	CHST2	ERICH6-AS1	MLF1
TPRG1	CCDC50	SPON2	TACC3	NSG1	RP11-539L10.2	CD38	FGFBP2	DTHD1	TXK
HOPX	IGFBP7	JCHAIN	CXCL8	PF4	PPBP	CXCL3	CXCL2	EREG	AREG
ARHGAP24	SNCA	DAPP1	BANK1	LEF1	PLA2G12A	44256	PALLD	HMGB2	HPGD
CASP3	BASP1	IL7R	DAB2	CCDC152	CTD-2035E11.3	GZMK	GZMA	KIF2A	F2R
VCAN	MEF2C	RHOBTB3	C5orf56	EGR1	MZB1	CXXC5	CD14	PPP2R2B	JAKMIP2
ADRB2	CSF1R	PDGFRB	SMIM3	SPARC	HAVCR2	PTTG1	DUSP1	RUFY1	F13A1
ADTRP	PHACTR1	GFOD1	GMPR	SOX4	RP11-367G6.3	HIST1H1C	HIST1H4C	HIST1H2AC	HIST1H4H
HIST1H2BJ	HIST1H3H	HIST1H2BN	IER3	LTB	LST1	NCR3	AIF1	C6orf25	HSPA1A
HSPA1B	HLA-DRA	HLA-DRB5	HLA-DRB1	HLA-DQA1	HLA-DQB1	HLA-DQA2	HLA-DOB	HLA-DMB	HLA-DMA
HLA-DPA1	HLA-DPB1	CDKN1A	TREML1	PTCRA	MAD2L1BP	ENPP5	MYO6	PRDM1	CD24
MARCKS	FAM26F	ARHGAP18	SAMD3	VNN2	TBPL1	SGK1	TNFAIP3	CITED2	STX11
RAB32	SYTL3	SOD2	HRAT92	TSPAN13	CPVL	MTURN	NT5C3A	AOAH	GPR141
NME8	STARD3NL	TRGC2	TRGC1	TRG-AS1	TRGV5	NCF1	FGL2	PTPN12	CD36
ABCB1	GNG11	BRI3	STAG3	PILRB	PILRA	NAMPT	PRKAR2B	SMKR1	RP11-138A9.2
TMEM140	TRBC1	TRBC2	TMEM176B	TMEM176A	LINC00685	IL3RA	AP1S2	SAT1	CYBB
TIMP1	CFP	PCSK1N	TSPYL2	RP6-159A1.4	CXCR3	NGFRAP1	PGRMC1	FHL1	PDZD4
TKTL1	MPP1	ARHGEF1	MYOM2	BLK	DMTN	SLC25A	ADAM28	DPYSL2	CLU

		0				37			
RP11-489E7.4	NRG1	PLPP5	CEBPD	TOX	RP11-25K19.1	MYBL1	MSC	TMEM70	LY96
CA2	TMEM55A	NCALD	KLF10	MYC	LYPD2	CDKN2A	B4GALT1	ENHO	CD72
RECK	TMEM2	ALDH1A1	PCSK5	CEP78	TLE1	GOLM1	RP11-305L7.1	RP11-305L7.3	CARD19
PTCH1	KLF4	UGCG	SLC31A2	RGS3	GSN	STOM	PTGS1	TTC16	CERCAM
NUP214	RP11-544A12.8	FCN1	PTGDS	CLIC3	C9orf139	NPDC1	IFITM3	IRF7	ASCL2
CD81	CDKN1C	OSBPL5	HBB	HBG2	ILK	RRAS2	PRR5L	SPI1	MPEG1
MS4A6A	MS4A7	MS4A1	FERMT3	PPP1R14B	AP003068.23	NEAT1	CTSW	CATSPER1	CTTN
FOLR3	AP001189.4	SYTL2	PRSS23	BIRC3	PDGFD	NCAM1	ZBTB16	ARHGEF12	CRTAM
NRGN	ESAM	FEZ1	SENCR	APLP2	B3GAT1	AKR1C3	IL2RA	RSU1	VIM-AS1
DNAJC1	OTUD1	MAP3K8	ZNF438	NCOA4	C10orf128	PRF1	PSAP	DDIT4	FUT11
VCL	IFIT3	HHEX	PDLIM1	PYROXD2	GSTO1	XPNPEP1	RGS10	PLEKHA1	CTBP2
CD9	CD27	ACRBP	PTMS	LAG3	SLC2A3	CLEC4A	CLEC4E	KLRG1	A2M-AS1
LINC00987	KLRB1	CLECL1	KLRF1	CLEC12A	CLEC1B	CLEC7A	GABARAPL1	KLRD1	KLRC4
KLRC2	KLRC1	RP11-291B21.2	YBX3	PLBD1	MGST1	PLEKHA5	KIF21A	RP11-446N19.1	TUBA1C
NFE2	RP11-620J15.3	IFNG	RP11-81H14.2	LYZ	RP11-1143G9.4	DUSP6	LINC00936	RP11-693J15.5	GNPTAB
CMKLR1	HVCN1	TESC	OASL	TMEM120B	BCL7A	LINC00944	RGCC	TSC22D1	RCBTB2
KCTD12	GPR183	TNFSF13B	IRS2	RNASE6	RNASE2	ARHGEF40	TRAV4	TRDC	SLC7A7
CMTM5	IRF9	GZMH	GZMB	NFKBIA	RP11-596C23.2	PTGDR	PTGER2	CGRRF1	LGALS3
RP11-902B17.1	PLEKHG3	MAX	ZFP36L1	ACTN1	NPC2	MLH3	FOS	RP11-7F17.8	GPR68
IFI27	SERPINA1	TCL1A	WARS	LINC00239	TNFAIP2	CKB	PLD4	CRIP2	IGHA2
IGHG4	IGHG2	AL928768.3	IGHA1	IGHG1	IGHG3	IGHD	IGHM	KIAA0125	APBA2
THBS1	PATL2	C15orf48	LINC00926	TPM1	DAPK2	KIAA0101	PIF1	NEIL1	BCL2A1
MCTP2	HBA2	HBA1	IL32	SOCS1	IL4R	GTF3C1	CD19	RP11-231C14.7	C16orf54
ITGAM	CES1	MT2A	MT1E	CCDC102A	ADGRG5	ADGRG1	KIFC3	TPPP3	TXNL4B
MAF	COTL1	IRF8	SERPINF1	CLUH	P2RX5	VMO1	CLEC10A	ASGR1	CD68
TMEM107	TNFRSF13B	RP11-160E2.6	RP11-47L3.1	CCL3	CCL4	AC131056.3	CCL3L3	CCL4L2	ERBB2
RP5-1028K	CCR7	GRN	ITGA2B	TBX21	COPZ2	HOXB4	RP11-357H	ABCC3	MMD

7.2							14.17		
RP11-758H9.2	CD79B	PECAM1	RGS9	KIF19	CD300A	LLGL2	MXRA7	CD7	RAB31
RAB27B	TCF4	PMAIP1	CD226	FAM110A	SMOX	CD93	CST3	ID1	BCL2L1
HCK	MYL9	SLA2	MAFB	PKIG	CTSA	CEBPB	LINC01272	NFATC2	TSHZ2
CTSZ	TUBB1	PRSS57	CFD	GNG7	MATK	TMIGD2	CD70	STXBP2	RETN
MCEMP1	FCER2	CD320	44257	S1PR5	C19orf38	JUNB	LYL1	IER2	CTD-3252C9.4
TPM4	HSH2D	FAM129C	IFI30	LRRC25	PLEKHF1	CD22	TYROBP	PPP1R14A	CAPN12
ZFP36	SERTAD1	BLVRB	TMEM91	CD79A	POU2F2	CNFN	PLAUR	FOSB	PPM1N
PTGIR	BBC3	FCGRT	SPIB	SIGLEC7	FPR1	ZNF600	LILRB2	LILRA5	LILRA4
LAIR2	KIR2DL3	KIR2DL1	KIR3DL1	KIR3DL2	CTB-61M7.2	NCR1	TNNT1	SYT5	RPS4Y1
DDX3Y	EIF1AY	CLDN5	YPEL1	IGLC2	IGLC3	VPREB3	DERL3	MIAT	GAS2L1
OSM	YWHAH	HMOX1	CSF2RB	IL2RB	LGALS2	MAFF	APOBEC3A	SYNGR1	GRAP2
TNFRSF13C	PARVB	PRR5	TTC38	MLC1	TYMP	ODF3B	MAP3K7CL	MX1	COL6A2
MCM3AP-AS1	DIP2A	S100B							

Supplementary Table 5

Subjects	Analysis method
Single cell sequencing	GemCode Single-Cell Instrument and Single Cell 3' Library & Gel Bead Kit v2 and Chip Kit (10x Genomics)
Cell counting	Cell ranger (version 2.2.0)
Expression calculation	Cell ranger (version 2.2.0)
UMI count matrix	R package Seurat (version 2.3.4)
Cell clustering	PCA and tSNE dimensionality clustering analysis
Unbiased cell type recognition	R package SingleR
Differentially expressed genes (DEGs) identification	Find Markers function in R package Seurat (version 2.3.4)
Generation of heatmaps, volcano plots, and violin plots	R software
Pathways analysis	Kyoto Encyclopedia of Genes and Genomes Database
Generation of Venn diagrams and Circoheat maps	TB tools
Pearson correlation coefficient calculation	R softeware
Generation of quantification histograms	GraphPad Prism software version 7.0
Statistical analysis on expression of DEGs	One-way analysis of variance (ANOVA) with Tukey's post hoc test
Genetic interaction analysis	String
Genetic relationship network	Cytoscape (Version 7.1)
